# Clinical and genetic spectrum of glycogen storage disease in Iranian population using targeted gene sequencing

**DOI:** 10.1038/s41598-021-86338-4

**Published:** 2021-03-29

**Authors:** Zahra Beyzaei, Fatih Ezgu, Bita Geramizadeh, Mohammad Hadi Imanieh, Mahmood Haghighat, Seyed Mohsen Dehghani, Naser Honar, Mojgan Zahmatkeshan, Amirreza Jassbi, Marjan Mahboubifar, Alireza Alborzi

**Affiliations:** 1 Shiraz Transplant Research Center (STRC), Shiraz University of Medical Sciences, Shiraz, Iran; 2 Department of Pediatric Metabolism and Genetic, Gazi University Faculty of Medicine, Ankara, Turkey; 3 Department of Pathology, Shiraz University of Medical Sciences, Khalili St., Research Tower, Seventh Floor, Shiraz Transplant Research Center (STRC), Shiraz, Iran; 4 Gastroenterology and Hepatology Research Center, Shiraz University of Medical Sciences, Shiraz, Iran; 5 Department of Pediatrics, Shiraz University of Medical Sciences, Shiraz, Iran; 6 Medicinal and Natural Products Chemistry Research Center, Shiraz University of Medical Sciences, Shiraz, Iran; 7 Student Research Committee, Shiraz University of Medical Sciences, Shiraz, Iran

**Keywords:** Genetics, Medical research, Molecular medicine

## Abstract

Glycogen storage diseases (GSDs) are known as complex disorders with overlapping manifestations. These features also preclude a specific clinical diagnosis, requiring more accurate paraclinical tests. To evaluate the patients with particular diagnosis features characterizing GSD, an observational retrospective case study was designed by performing a targeted gene sequencing (TGS) for accurate subtyping. A total of the 14 pediatric patients were admitted to our hospital and referred for molecular genetic testing using TGS. Seven genes namely *SLC37A4*, *AGL*, *GBE1*, *PYGL*, *PHKB*, *PGAM2*, and *PRKAG2* were detected to be responsible for the onset of the clinical symptoms. A total number of 15 variants were identified i.e. mostly loss-of-function (LoF) variants, of which 10 variants were novel. Finally, diagnosis of GSD types Ib, III, IV, VI, IXb, IXc, X, and GSD of the heart, lethal congenital was made in 13 out of the 14 patients. Notably, GSD-IX and GSD of the heart-lethal congenital (i.e. PRKAG2 deficiency) patients have been reported in Iran for the first time which shown the development of liver cirrhosis with novel variants. These results showed that TGS, in combination with clinical, biochemical, and pathological hallmarks, could provide accurate and high-throughput results for diagnosing and sub-typing GSD and related diseases.

## Introduction

Glycogen storage diseases (GSDs) are known as a group of disorders characterized by genetic errors leading to accumulation of glycogen in various tissues^1^. All the main types of GSD that are currently recognized primarily affect the liver and/or muscles as the main organs of involvement^2^. However, accurate identification of the sub-types of GSDs, especially hepatic forms, is not an easy task for clinicians and pathologists because of their similar and overlapping features as well as wide phenotypic variations^3^.

It is known that early diagnosis is important for proper treatment of patients to decrease organ damage and to increase life expectancy^4^. The diagnosis of GSDs mostly depends on paraclinical, clinical and biochemical assays^5^. Molecular analysis, based on DNA testing, similarly permits accurate diagnosis when enzymological and pathological results are equivocal or unavailable^6^. Identification of GSD patients’ genetic background along with mutation screening can provide the best approach to diagnosis and classification^7^.

NGS has been applied in clinical diagnostics for a diversity of symptoms to characterize the inherent genetic cause of diseases^8^. Although single-gene testing and gene panels for specific disorders are still being used, NGS is progressively being utilized in diagnostic evaluation, especially for disorders that are genetically heterogeneous, such as GSDs^9,10^. Currently, targeted gene sequencing (TGS) panels have gained popularity for heterogeneous genetic anomalies in monogenic disorders (MDs) because of their time- and cost-effectiveness as well as their ability in simultaneous detection of common and rare genetic variations^11^. According to the aforementioned background, the present study aimed to identify the genetic background of GSDs in a small sample of Iranian patients by using targeted gene sequencing (TGS) to search for molecular etiology. To the best of our knowledge, this is the first study from Iran.

## Results

### Demographic characteristics of the patients

A total number of 14 pediatric patients were recruited in this retrospective observational case study. There were particular diagnosis features leading to their selection as GSD patients. Among them, six cases (42.8%) were male and eight (57.2%) were female. Parents of 13 patients (86.7%) were consanguineous, five of them with a family history of liver diseases from early infancy. The mean age of the disease onset was 14.1 months (range: 1–35) and average delay to establish an accurate diagnosis was 33.4 months (range: 14–51). Short stature (< 3%) was also observed in eight patients. High triglyceride (TG), total cholesterol (TChol), and lactate dehydrogenase (LDH) were observed in nine (64%), nine (64%), and eight patients (57%), respectively. High creatine phosphokinase (CPK) and platelet count was detected in five patients (36%) and low uric acid was observed in one patient (7%). High blood urea nitrogen (BUN)/creatinine ratio (BCR) was additionally detected in eight patients (57%). Elevated liver enzymes, i.e., aspartate aminotransferase (AST), alanine aminotransferase (ALT), and hepatomegaly, were observed in all patients except one. All clinical manifestations are summarized in Table 1.

**Table 1:** Clinical manifestations of Iranian patients.

Patients /gender/GSD type	Onset (month)	Clinical indication	Marriage type	Short stature (< 3%)	TG^a^ (mg/dL)	T.Chol^a^ (mg/dL)	LDH^a^ U/L	BCR	Ph	Uric acid^b^ (mg/dL)	CPK^c^ (U/L)	Platelet^d^ (10*3/µL)	Alb^e^ (g/dL)	AST^f^ (U/L)	ALT^f^ (U/L)
P1/F/Ib	1	Hypoglycemia, hepatomegaly , and low WBC, high RBC and platelets	Consanguineous	–	709	139	347	–	Acidic	5.5	35	529	3.8	121	78
P2/F/GSD III	23	Hypoglycemic seizures at age 2, hepatomegaly	Consanguineous	Yes	584	279	1950	17.5	–	4.5	190	400	4.6	3300	1310
P3/M/III	9	Hepatomegaly, hypoglycemia	Consanguineous	Yes	336	231	1540	32.5	–	4.8	202	411	4.3	492	693
P4/F/III	9	Hepatomegaly, hypoglycemia	Consanguineous	Yes	–	–	754	27.5	–	4	1120	470	4.3	382	502
P5/M/III	18	Hepatomegaly, abdominal Protrusion	Consanguineous	No	639	360	841	35	–	4.7	585	283	4.2	308	568
P6/M/IV	16	Hepatomegaly, enlargement of spleen	Consanguineous	No	102	149	60	28.9	–	5.5	370	480	4.3	199	114
P7/F/VI	24	Hepatomegaly	Consanguineous	Yes	223	98	590	48	Acidic	3.8	129	397	4.4	78	182
P8/M/VI	12	Hepatomegaly, Abdominal Protrusion, malaise	Consanguineous	No	144	159	–	45	–	–	118	461	4.2	141	172
P9/F/IXc	6	Hepatomegaly, hypoglycemia	Non-consanguineous	Yes	160	141	504	12.5	–	–	141	–	4.2	318	173
P10/M/IXb	8	Hepatomegaly	Consanguineous	Yes	156	218		25	–	–	342	331	4.4	660	600
P11/F/Ib, IXb	35	Asymptomatic ( poor feeding), low WBC	Consanguineous	No	498	313	600	20	Acidic	5.8	–	201	4.4	520	466
P12/M/X	21	Mild hepatomegaly, low creatinine	Consanguineous	No	401	279	584	50	acidic	–	–	370	4.7	606	643
P13/F/glycogen storage disease of heart, lethal congenital	1	Hepatomegaly	Non-consanguineous	Yes	66	195		20	–	–	18	221	3.8	28	11
P14/F/NA	20	Hepatomegaly, FTT, diarrhea, vomiting, high platelet	Consanguineous	Yes	126	154	414	12.5	Acidic	1.5	–	477	4.1	136	107

*GSD* glycogen storage disease, *FTT* failure to thrive, *TG* triglyceride, *Chol* cholesterol, *BCR* blood urea nitrogen (BUN)/creatinine ratio, *Alb* albumin, *ALT* alanine transaminase, *AST* aspartate transaminase, *CPK* creatine phosphokinase.

^a^Reference range for TG, Total chol < 150 mg/dL; LDH < 480 U/L.

^b^Reference normal range for uric acid 3.5–8.2 mg/dL.

^c^Reference normal range for CPK 24–195 U/L.

^d^Reference normal range for Platelet 145–450 10*3/µL.

^e^Reference normal range for Alb = 3.5–5.4 g/dL.

^f^Reference range for liver enzymes: ALT < 40 U/L; AST < 45 U/L.

### Targeted gene sequencing (TGS) data

In order to identify the molecular etiology, TGS was performed using the patients’ peripheral blood. A total of 450 genes of inherited metabolic diseases were included in this panel. All coding regions for the 450 genes were enriched in an unbiased fashion, with sufficient coverage. The analysis was successful with 100% reads on target, 100 × coverage of 99.99% and 20 × coverage of 99.99%. Mean coverage of the targeted regions was 144 × per sample, (ranged: 116 × to 178 ×). Each patient showed an average 1200 sequence variants. All the variants were identified by TGS, confirmed by Sanger sequencing for each patient (Supplementary Table 1). Both the sensitivity and the specificity for base calls were 100% for the comparison with the results of Sanger sequencing of the same set of samples. Finally, the results showed to be concordant in terms of zygosity.

### Genomic diagnostic results

Diagnoses and zygosity of the 14 patients are illustrated in Table 2. Pathogenic or novel variants in different GSD associated genes were detected in 13 out of 14 patients (93%). Accordingly, one patient (6.7%) had GSD-I, four (26.6%) were affected with GSD-III, one (6.7%) had GSD-IV, two cases (13.3%) were suffering from GSD-VI, three patients (20%) had GSD-IX, one case was affected with (6.7%) GSD-X, and one patient (6.7%) was suffering from GSD of heart—lethal congenital disorder. Overall, 15 mutations were detected in the GSD-associated genes in 13 patients, 10 of whom had not been previously reported. These novel mutations included one frameshift variant in *AGL* (c.1351_1355delAAAGC), one nonsense change in *SLC37A4* (c.24T > G), and one splicing mutation (c.1127-2A > G) in *PHKB*. Moreover, there were seven missense variants, i.e. one in *PGAM2* (c.130C > T), one in *PYGL* (c.1964A > G), two in *PHKB* (c.134T > A; c.2840A > G), one in *PRKAG2* (c.592A > T), one in *SLC37A4* (c.337C > T), and one in *GBE1* (c.292G > C) (Table 2). Two patients were also detected to have bi-allelic mutations; patient no. 6 had mutations in *GBE1* gene, and patient no. 11 had mutations in two different genes, i.e., *SLC37A4* and *PHKB* (Table 2). The most common defects were found in *AGL* (GSD-III) and *PHKB* (GSD-IX). Allele frequency of all variants were searched in Iranome database (public Iranian data set). Only 13.3% of novel variants were observed in this database (which is rare with an allele frequency less than 0.001), as presented in Table 2. Finally, the diagnostic rate for TGS in patients suspected with GSD was 93% (13/14).

**Table 2:** Summary of GSD mutations detected by MPS-GSD panel.

Patients /gender/GSD type	Age at genetic diagnosis	Gene/inheritance pattern^a^	Chr: loc (hg19)	Nucleotide change	Predicted protein change	Variant type	Zygosity	Feature of liver histopathology	Previous definition and pathogenicity	Iranome database
P1/F/Ib	14 mo	*SLC37A4/*AR	11: 118900056	**c.24T** **>** **G**	**p.Tyr8Ter**	Nonsense	Homozygous	GSD I with severe fibrosis, cirrhosis	Not defined, Pathogenic	NA
P2/F/III	41mo	*AGL/*AR	1: 100336041	c.753_756delCAGA	p.Asp251GlufsTer23	In-frame deletion	Homozygous	GSD I or III with early septal cirrhosis	Defined in HGMD, pathogenic	NA
P3/M/III	29 mo	*AGL/*AR	1: 100336041	c.753_756delCAGA	p.Asp251GlufsTer23	In-frame deletion	Homozygous	GSD I or III with mild portal fibrosis	Defined in HGMD, pathogenic	NA
P4/F/III	47 mo	*AGL/*AR	1: 100342081	**c.1351_1355delAAAGC**	**p.Lys451LeufsTer14**	Frame shift	Homozygous	GSD I or III with severe fibrosis	Not defined, pathogenic	NA
P5/M/III	36 mo	*AGL/*AR	1: 100379113	c.3980G > A	p.Trp1327Ter	Nonsense	Homozygous	GSD I or III with cirrhosis	Defined in HGMD, pathogenic	NA
P6/M/IV	51 mo	*GBE1/*AR	3: 81643169 3: 81754616	c.998A > T **c.292G** **>** **C**	p.Glu333Val **p.Val98Leu**	Missense Missense	Homozygous Homozygous	GSD IV with cirrhosis	Defined in HGMD, pathogenic Not defined, uncertain significance	NA NA
P7/F/VI	48 mo	*PYGL/*AR	14: 51378453	**c.1964A** **>** **G**	**p.Glu655Gly**	Missense	Homozygous	Unclassified GSD with marked fibrosis	Not defined, uncertain significance	NA
P8/M/VI	19 mo	*PYGL/*AR	14: 51410891	c.229_231delGAC	p.Asp77del	Deletion	Homozygous	GSD I or III with fibrosis	Defined in HGMD, pathogenic	NA
P9/F/IXc	28 mo	*PHKG2/*AR	16: 30762461	c.130C > T	p.Arg44Ter	Nonsense	Homozygous	Unclassified GSD with fibrosis	Defined in HGMD, pathogenic	NA
P10/M/IXb	36 mo	*PHKB/*AR	16: 47531367	**c.134T** **>** **A**	**p.Leu45His**	Missense	Heterozygous	Unclassified GSD with bridging fibrosis	Not defined, uncertain significance	0.0025
P11/F/Ib, IXb	41 mo	*SLC37A4/*AR *PHKB/*AR *PHKB/*AR	11: 118898407 16: 47628046 16: 47727384	**c.337C** **>** **T** **c.1127-2A** **>** **G** **c.2840A** **>** **G**	**p.Leu113Phe** **p.?** **p.Gln947Arg**	Missense Potential splice site Missense	Heterozygous Homozygous Homozygous	Unclassified GSD with moderate periportal fibrosis	Not defined, uncertain significance Not defined, likely pathogenic Not defined, uncertain significance	0.003125 NA NA
P12/M/X	29 mo	*PGAM2/*AR	7: 44105115	**c.14G** **>** **A**	**p.Arg5His**	Missense	Heterozygous	Unclassified GSD with early septal cirrhosis	Not defined, uncertain significance	NA
P13/F/GSD of heart, lethal congenital	29 mo	*PRKAG2/*AD	7: 151329185	**c.592A** **>** **T**	**p.Met198Leu**	Missense	Heterozygous	Unclassified GSD with cirrhosis	Not defined, uncertain significance	NA
P14/F/NA	28 mo	NA	–	None in GSD or similar phenotype genes	–	–	–	Unclassified GSD or lipid storage disease with mild portal fibrosis	–	–

Bold type represents novel unclassified variants.

*AD* autosomal dominant, *AR* autosomal recessive, *Chr Loc* chromosome location, *F* female, *GSD* glycogen storage disease, *HGMD* human gene mutation database, *M* male, *mo* month, *P* patient, *VUS* variant of unknown significance.

^a^The Accession Number of the relevant reference sequence(s): SLC37A4, RefSeq NM_001164279.2; AGL, Ref Seq NM_000642.3; GBE1, RefSeq NM_000158.4; PYGL, RefSeq NM_002863.5; PHKG2, RefSeq NM_000294.3; PHKB, RefSeq NM_000293.3; PGAM2, RefSeq NM_000290.4; PRKAG2, RefSeq NM_001040633.1.

### Comprehensive analysis for detection of variants in GSD patients

*Patient no. 1* was a 1-year-old girl who presented with hypoglycemia, hepatomegaly, elevated TG, acidic urine, platelet count, and low white blood cells (WBCs) from a consanguineous marriage, suggestive of GSD-I (Table 1). Pathological results also indicated GSD-I with severe bridging fibrosis, diagnosed as cirrhosis. A novel homozygous nonsense variant, i.e., c.24T > G (p.Tyr8Ter), was also detected in the *SLC37A4* gene (GSD type-Ib) by TGS (Table 2). No other deleterious variant was found in other GSD genes in the panel.

*Patients no. 2–3* had clinicopathological and histochemical findings, strongly suggestive of GSD-I or III. Both patients were presented with hypoglycemia (patient no. 2 also had experienced seizures at the age of 2), hepatomegaly, short stature, elevated TG, TChol, LDH, albumin (Alb), AST, and ALT enzymes (Table 1). Histopathological findings were suggestive of type I or III GSD, with mild portal fibrosis. The variants in the glycogen debranching enzyme gene, *AGL,* were also observed by TGS. A homozygous deleterious frameshift mutation, i.e. c.753_756delCAGA (p.Asp251fs*23), was further detected in the *AGL* gene in both patients, which had been previously reported in patients affected with GSD-IIIa^12^.

*Patient no. 4* was a 4-year-old girl with hypoglycemia, hepatomegaly, short stature, elevated LDH, CPK, platelet count, AST, and ALT, whose parents were first cousins. The liver biopsy from this case suggested GSD-I or III along with severe fibrosis. A novel pathogenic homozygote variant, c.1351_1355delAAAGC (p.Lys451LeufsTer14), was also detected in the *AGL* gene. This variant had not been listed in Iranome and gnomAD databases or described in the related literature.

Another example of GSD-III was *patient 5*, a 3-year-old boy, who presented with hepatomegaly, elevated TG, TChol, LDH, BCR, AST, and ALT. The liver biopsy diagnosis in this case was GSD-I or III with cirrhosis. The targeted NGS also detected a homozygote variant, c.3980G > A (p.Trp1327Ter), which had been previously reported^13,14^.

*Patient no. 6* was a 4.5-year-old boy with clinical and paraclinical findings such as hepatosplenomegaly, as well as elevated BCR, AST, and ALT (Table 1). In addition, the liver biopsy showed cirrhosis and suggested GSD-IV. He had also successfully received a partial liver transplant at the age of 2. Moreover, the targeted NGS panel revealed two variants in *GBE1* gene. A homozygous deleterious variant, namely c.998A > T (p.Glu333Val)^15^, and another novel homozygous variant c.292G > C (p.Val98Leu), were additionally detected in the *GBE1* gene. The new variant was not listed in Iranome and gnomAD databases or described in the related literature, so it could be interpreted as a variant of uncertain significance (VUS).

*Patient no. 7* was a 4-year-old girl, presented with hepatomegaly, short stature, high TG, LDH, BCR, Alb, AST, ALT, and acidosis (Table 1). Her liver biopsy also suggested unclassified GSD with marked fibrosis. Using TGS, a novel homozygous missense variant, c.1964A > G (p.Glu655Gly), was detected in *PYGL* gene, indicating GSD-VI (Table 2).

*Patient no. 8* was a 1.5-year-old boy, referred with hepatomegaly, abdominal protrusion, and malaise (Table 1). Para-clinical results also showed increased TG, TChol, BCR, AST, and ALT (Table 1). Histopathological studies of his liver biopsy also suggested GSD-I or III with mild fibrosis. However, a homozygous pathogenic deletion variant, c.229-231delGAC (p.Asp77del), was detected in the liver isoform glycogen phosphorylase, the *PYGL* gene (Table 2)^16^.

*Patient no. 9* was a 2-year-old girl from a non-consanguineous marriage with episodes of hypoglycemia starting from six months of age during nighttime, hepatomegaly, short stature, elevated AST, and ALT (Table 1). Her liver biopsy also showed unclassified GSD with fibrosis. A homozygous pathogenic variant, c.130C > T (p.Arg44Ter), was additionally detected in a *PHKG2* gene by TGS. This missense mutation had been previously reported in patients with GSD-IXc^17–19^.

*Patient no. 10* was a 3-year-old boy, who presented with hepatomegaly, short stature, and muscular hypotonia as well as elevated TG, LDH, TChol, AST, and ALT since the age of six months (Table 1). The results of liver histopathological studies also showed unclassified GSD with bridging fibrosis. Using TGS analysis additionally revealed a novel heterozygous variant, c.134T > A (p.Leu45His), in the glycogen phosphorylase kinase regulatory sub-unit beta gene, *PHKB* (GSD-IXb). No other pathogenic variants were detected in other GSD genes in the panel.

*Patient no. 11* was an asymptomatic girl whose parents were first cousins. She was referred because of poor feeding at the age of 3. Laboratory investigations also showed elevated TG, TChol, LDH, Alb, AST, and ALT, as well as leukopenia and acidosis (Table 1). The liver biopsy revealed unclassified GSD, and moderate periportal fibrosis. She harbored three novel variants, namely one heterozygote variant c.337C > T (p.Leu113Phe) in the *SLC37A4* gene and two homozygote variants c.1127-2A > G (p.?) and c.2840A > G (p.Gln947Arg) in the *PHKB* gene. The pathogenic novel variant, c.1127-2A > G (p.?), was possibly damaging the splice site located within intron. As a result, she was most probably suffering from IXb, whose symptoms tended to appear with increasing age. Moreover, targeted NGS successfully identified these three mutations with 100 × coverage.

*Patient no. 12* was a 2.5-year-old boy with mild hepatomegaly, high TG, TChol, LDH, BCR, AST, and ALT enzyme and very low creatinine (Table 1). Histopathological studies of his liver biopsy also suggested unclassified GSD, with cirrhosis. Using TGS, a novel heterozygous variant, c.14G > A (p.Arg5His), was detected in phosphoglycerate mutase gene, the *PGAM2* (GSD-X). To note, GSD-X is an autosomal recessive disorder and the detection of a single heterozygous variant did not confirm the diagnosis. Nevertheless, lack of a second pathogenic allele or any identified pseudo-deficiency variant had left the molecular diagnosis of this patient in question. The signs may be caused by pathogenic variants in other genes including disorders of fatty acid oxidation and/or mitochondrial respiratory chain disorders.

*Patient no. 13* was a 2.5-year-old girl who presented with short stature and normal biochemical analysis of a non-consanguineous marriage (Table 1). Pulmonary hypertension, moderate mitral regurgitation, and mild tricuspid regurgitation were also observed. Moreover, the liver biopsy results revealed cirrhosis, which was suggestive of unclassified GSD. A novel heterozygous variant, c.592A > T (p.Met198Leu), was further detected in the *PRKAG2* gene by TGS and implied *PRKAG2* deficiency (i.e. GSD of heart—lethal congenital). Since the *PRKAG2* deficiency is an autosomal dominant inheritance with full penetrance, single heterozygote variants could confirm all of her clinical, molecular, and biochemical results.

The diagnosis of none of the GSD and non-GSD-associated genes was confirmed in *patient no. 14*. She was a 2-year-old girl, who presented with hepatomegaly, clubbed fingers, failure to thrive, diarrhea, vomiting, as well as high platelet count, AST, ALT and low uric acid (Table 1). Her liver biopsy was suggestive of GSD or lipid storage disease with mild fibrosis. No deleterious mutations were also detected in any of the related GSD genes analyzed. There was, therefore, no definite diagnosis for this patient.

### Histological findings and association with genetic sequencing

In five patients, the features of liver histopathology were suggestive of unclassified GSD, molecular genetic investigations of these patients which confirmed the diagnosis of GSD-VI in one patient (no. 7), GSD-IXb in two cases (no. 10 and 11), diagnosis of GSD-IXc (no. 9), and diagnosis of GSD of heart—lethal congenital disorder—in one patient (no. 3). In one case, not only the features of liver histopathology were shown ambiguous results, but also no deleterious mutations were detected in any of the GSD genes analyzed (no. 14).

Among the nine calculated pathogenic variants identified in our cohort, we identified eight cases (88%) to have severe fibrosis/ cirrhosis. On the other hand, one case (12%) of VUS showed severe fibrosis/ cirrhosis in liver biopsy. Therefore, there was a significant association between the pathogenicity of the variants and the features of liver histopathology in the patients, as presented in Table 3 (*P* = 0.049).

**Table 3:** An evaluation of the identified variation in molecular results and pathology investigations.

Pathology	Molecular results*		*P* value**
	Pathogenic variant	VUS	
Fibrosis/cirrhosis	8	1	
No fibrosis/cirrhosis	1	3	
Total	9	5	0.049

*In total of 13 patients, one patient was undiagnosed with TGS.

**P associated with Fisher's exact test.

## Discussion

Classification and sub-typing of GSD patients are important steps towards personalized patient management, which can help clinicians practice the best and the most correct therapy with the fewest adverse events for patients^20^. Here, the first and largest cohort is reported about GSD sub-typing from the Middle East and Asia. It is also the first study, addressing clinical characteristics and genomics in sub-typing of patients with GSDs from Iranian population. In this cohort of 14 pediatric patients, 10 novel pathogenic variants in the *SLC37A*, *AGL*, *GBE1*, *PHKB*, *PGAM2* and *PRKAG2* genes were found. In our patient cohort, the most common subtype was GSD III (27%). Notably, GSD-IX was detected in three patients, which had not been reported from Iran, so far. Concerning GSD-IX patients, the estimated prevalence is 1:100,000 and they account for 25–30% of all GSD cases^21,22^. It means that it has been overlooked in our population because of subtle patient presentations and self-limited outcomes as well as lack of molecular diagnosis analyses. Therefore, it has been classified as other types of GSD, such as GSD-III or VI.

Chronic liver diseases, such as cirrhosis and fibrosis, have been also rarely reported in some types of GSDs e.g. GSD-VI and IX^21^. However, in the present study, 40% of the patients had liver cirrhosis and 60% had different degrees of liver fibrosis. In addition, asymptomatic heart problems with liver involvement were identified in a GSD of the heart-lethal congenital disorder (i.e. PRKAG2 deficiency) in one patient in our study cohort. To the best of our knowledge, we report for the first time liver cirrhosis in GSD-X and GSD of the heart-lethal congenital (i.e. PRKAG2 deficiency). In this pathological report, 13 patients were suggestive to have one type of GSD without exact sub-typing, so molecular genetic analysis (namely, targeted genome sequencing based on NGS) was performed, confirming the exact type of GSD. According to these results, molecular genetic testing, especially NGS-based GSD or inborn inherited metabolic panel exome sequencing, was recommended for definite diagnosis of GSD sub-types prior to invasive liver biopsy. Liver histopathology may also be a powerful and effective method for monitoring long-term liver complications and evaluating the status of the liver in these patients, but not for confirming diagnosis and accurate sub-typing.

**Figure 1:**
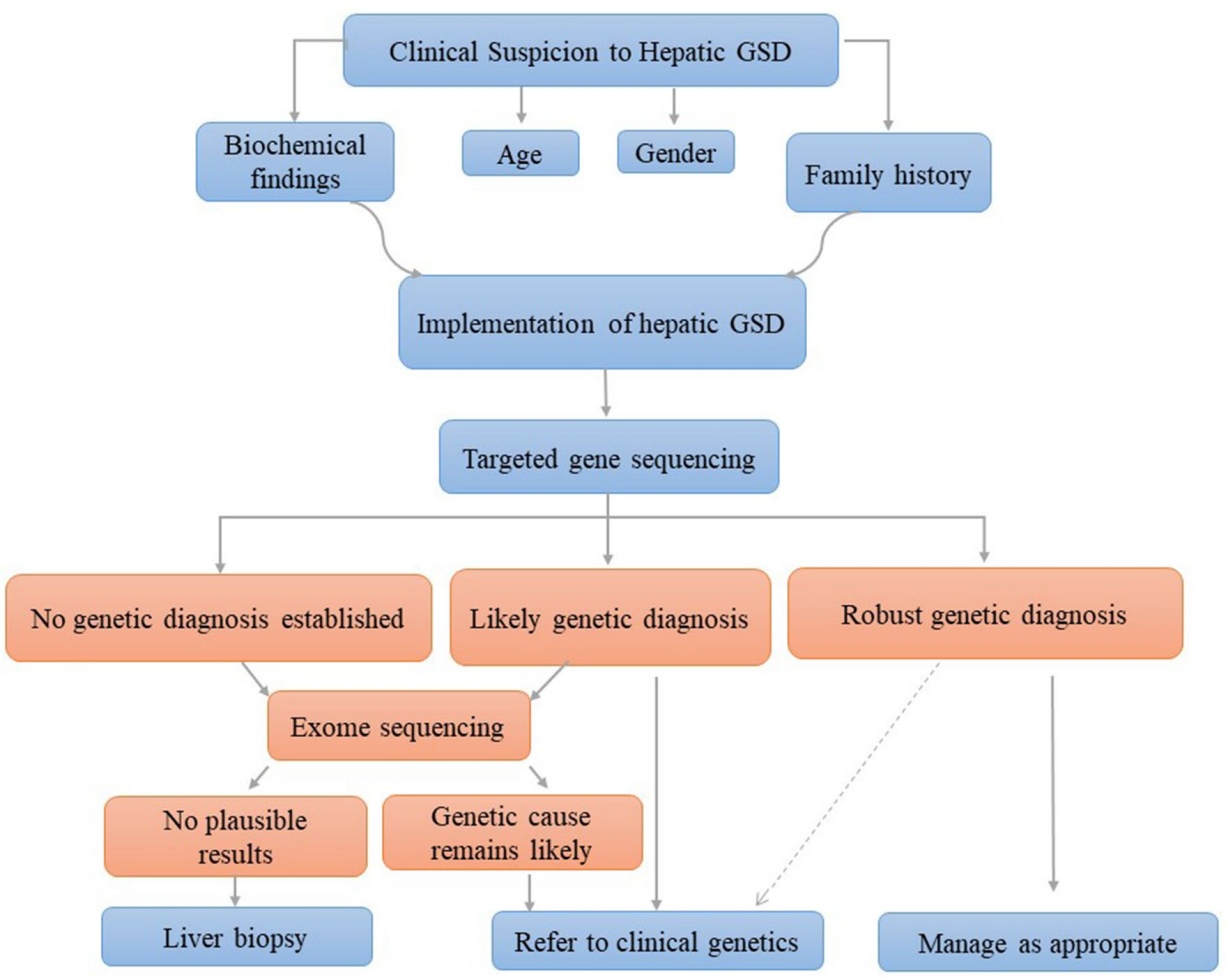
Integration of clinical and laboratory workflows to optimize hepatic glycogen storage disease diagnosis^24^.

NGS-based targeted exome sequencing is thus reported as the best future routine method of molecular diagnosis. This is especially useful for complex disorders with less specific clinical findings^23^. Nevertheless, in defining the syndromes or diseases like GSD, clinical features or biochemical phenotypes can effectively address a particular pathway or a group of genes responsible for the disease. In such cases, a custom-targeted gene-sequencing panel has been confirmed to be an efficient as well as time- and cost-effective technique with high diagnostic yields^24^. Analytical workflows for the diagnosis of GSD diseases are not fully standardized; however, a useful and practical approach based on clinical and biochemical evaluations followed by targeted molecular analysis was reported later, as shown in Fig. 1^24^. Moreover, using custom-target sequencing vs. exome sequencing would become a routine technique due to the focus on a limited number of suspected diseases and appropriate balance between the cost, time, throughput, and deep coverage, especially for low-income countries such as Iran^25^. To note, utilizing TGS panel is suitable to detect mutations, especially in communities with high numbers of consanguineous marriages such as Iran. In this country, the prevalence rate of consanguineous marriage is approximately seen in 38.6% of the population with a mean inbreeding coefficient (alpha) of 0.018, probably resulting in a higher incidence of autosomal recessive diseases such as GSDs^26^. Moreover, the samples from patients without a definite diagnosis would be recommended to be analyzed by genome sequencing or exome sequencing.

The present work revealed unexpected findings for two patients. Patient no.13 carried mutations associated with *PRKAG2* gene, which also developed liver failure. However, in previous studies, reported manifestations had been less severe and essentially heart-specific, non-lysosomal glycogenosis, and mild-to-severe cardiac hypertrophy, enhancing the risk of sudden cardiac death in midlife without liver involvement^27,28^. This was the first patient with *PRKAG2* gene mutation reported to have liver cirrhosis; however, a functionality of the novel variant remains underdiagnosed. Another patient (no. 14) showed liver problem and all similar clinical features to GSD; nevertheless, it was not possible to match it with any variant in the custom panel of inborn errors of metabolism. These two patients had atypical clinicopathological features, precluding accurate classification and diagnosis with clinicopathological features and in need of more specific genetic testing for definite diagnosis.

Despite genetic homogeneity, we found evidence of unusual features with novel variants. A possible reason for the high rate of novel variations we saw might be the lack of molecular genetic analysis before. It is known that mutations can have a specific race as well as restricted geographical or ethnical distribution, while was never analyzed such patients in our country. In addition, the results of this study will help improve gene variant spectrum, diagnostic panels, clinical diagnosis, and patient management not only in this country but also in the region. A deeper knowledge of genomic variants also leads to better findings of determinants associated with the genotype–phenotype match in GSDs^29^.

In conclusion, the study indicated the benefits of TGS method in diagnosing GSD, especially when the clinical findings were equivocal. Given the cost- and time-efficiency of these methods, they can prevent the patients from receiving long-term improper treatments. The diagnosis of the patients reported here has helped expand the genetic and phenotypic spectrum of the GSDs disorders.

## Materials and methods

### Participants

From March 2017 to December 2019, a total number of 14 pediatric patients suspected to GSDs who presented with hepatomegaly, hypoglycemia, growth and development delay during childhood were selected at Shiraz Transplant Research Center (STRC) and Namazi Hospital (Shiraz, Iran). None of these 14 cases had molecular diagnoses. All the patients had already have liver biopsies with histopathological features, which suggested hepatic GSDs by the pathologist (Liver biopsy was performed to determine the details of the liver pathology especially stage of fibrosis). Two independent research team members reviewed electronic and paper charts for clinical features, biochemical investigations, histopathological results, and diagnostic imaging. Whole blood samples were collected from all study subjects and sent to the Pediatric Metabolic Diseases Laboratory, Gazi Hospital (Ankara, Turkey) for targeted NGS-based panel analysis. To this end, the subjects’ parents/guardians signed written informed consents. The Ethics Committee of Shiraz University of Medical Sciences also approved this study (Approval #: IR.SUMS.REC.1396.S805), which was in accordance with the Declaration of Helsinki.

### Gene panel sequencing

In brief, genomic DNA from 2 ml peripheral blood was extracted using AutoMate Express Nucleic Acid Extraction System (Life Technologies, Guilford, CT, South San Francisco, CA, US). They were also hybridized and enriched for TGS. Then, Ion Torrent S5 platform was employed for DNA sequencing analysis. A custom-targeted Ion AmpliSeq panel that included 7219 amplicons covering 450 genes associated with Inborn Metabolic Diseases was used. Among 450 genes, the GSD genes were also present in this panel which included the genes for Glycogen Storage Disorders with hepatic involvement such as *G6PC* (Type Ia), *SLC37A4* (Type Ib), *AGL* (Type III), *GBE1* (Type IV), *PYGL* (Type VI), *PHKA2* (Type IXa), *PHKB* (Type IXb), *PHKG2* (Type IXc) and *GLUT2* (Type XI). The other genes for gluconeogenesis, namely *PC* (Pyruvate Carboxylase deficiency), *PCK2* (Phosphoenolpyruvate carboxykinase deficiency) and *FBP1* (Fructose-1,6-bisphosphatase), were also present in this panel.

The panel similarly covered 3′ untranslated regions (UTRs) of the genes and extended 5 bp on either side of each exon (Life Technologies, Guilford, CT, South San Francisco, CA, US). Analyses were done using an Ion Torrent 540 chip (Life Technologies, Guilford, CT, South San Francisco, CA).

The results were analyzed with Ion Reporter Software (Life Technologies, Guilford, CT, South San Francisco, CA, US) as well as Integrated Genomic Viewer^30^. The human genome 19 was also used as the reference. Polymorphism Phenotyping v2 (PolyPhen2), Scale-Invariant Feature Transform (SIFT), and MutationTaster were further employed for in silico analysis. Genomic Evolutionary Rate Profiling (GERP) and the Phastcons scores were also utilized to evaluate the conservation of the variants. The population frequency of each variation was correspondingly estimated using the data from the Genome Aggregation Database (gnomAD) and Iranome database^31^. The American College of Medical Genetics and Genomics (ACMG) guidelines were additionally used for variant interpretations^32^. The sequence variants were also described according to the Human Genome Variation Society Nomenclature^33^. Accession number of the relevant reference sequence(s) of GSD genes are presented in Supplementary File 1.

### Validation of candidate genes

Direct Sanger sequencing was performed in all subjects for validation of the causal mutations in candidate genes. Primers were designed using OLIGO primers design v.7 (Molecular Biology Insights, Inc., DBA Oligo, Inc.) which were sequenced by standard Sanger’s sequencing technique using BigDyeTerminator (Invitrogen, ABI, Foster City, CA).

### Liver biopsy

All patients had undergone ultrasound-guided liver biopsy using the standard Tru-Cut biopsy needles. Histopathology slides were prepared and stained routinely by Hematoxylin and Eosin (H&E), Periodic acid-Schiff (PAS), PAS with diastase (PAS + D), Trichrome, Reticulin, and Iron staining. All the slides were reviewed by an expert hepatopathologist (B.G.).

### Statistical analysis

Data were analyzed using SPSS 16.0 for Windows (SPSS Inc., Chicago, IL, USA). Continuous data were presented as the mean and standard deviation (SD) or median and range. The Fisher’s exact test was used to compare the relationship between the liver pathogenesis and pathogenic variant presence. A two-tailed value for *P* < 0.05 was considered statistically significant.

### Ethics approval

All procedures performed in studies involving human participants were in accordance with the ethical standards of the institutional and/or national research committee and with the 1964 Helsinki Declaration and its later amendments or comparable ethical standards. The study was approved by the Bioethics Committee of the Medical University of Shiraz, Iran (No. IR.SUMS.REC.1396.S805).

### Consent for publication

Informed consent was obtained from legal guardians.

## Supplementary Information

Supplementary Information.

## Data Availability

The data that support the findings of this study are available on request from the corresponding author. The data are not publicly available due to privacy and ethical restrictions.

## Abbreviations

GSD: Glycogen storage disease
MPS: Massively parallel sequencing
NGS: Next-generation sequencing
TGS: Targeted genome sequencing
VUS: Variant of unknown significance
