# Author Correction: Clinical and genetic spectrum of glycogen storage disease in Iranian population using targeted gene sequencing

**DOI:** 10.1038/s41598-021-94296-0

**Published:** 2021-07-26

**Authors:** Zahra Beyzaei, Fatih Ezgu, Bita Geramizadeh, Mohammad Hadi Imanieh, Mahmood Haghighat, Seyed Mohsen Dehghani, Naser Honar, Mojgan Zahmatkeshan, Amirreza Jassbi, Marjan Mahboubifar, Alireza Alborzi

**Affiliations:** 1 Shiraz Transplant Research Center (STRC), Shiraz University of Medical Sciences, Shiraz, Iran; 2 Department of Pediatric Metabolism and Genetic, Gazi University Faculty of Medicine, Ankara, Turkey; 3 Department of Pathology, Shiraz University of Medical Sciences, Khalili St., Research Tower, Seventh Floor, Shiraz Transplant Research Center (STRC), Shiraz, Iran; 4 Gastroenterology and Hepatology Research Center, Shiraz University of Medical Sciences, Shiraz, Iran; 5 Department of Pediatrics, Shiraz University of Medical Sciences, Shiraz, Iran; 6 Medicinal and Natural Products Chemistry Research Center, Shiraz University of Medical Sciences, Shiraz, Iran; 7 Student Research Committee, Shiraz University of Medical Sciences, Shiraz, Iran

Correction to: *Scientific Reports* 10.1038/s41598-021-86338-4, published online 29 March 2021

The original version of this Article contained an error in the Abstract.

“A total of the 15 pediatric patients were admitted to our hospital and referred for molecular genetic testing using TGS. Eight genes namely *SLC37A4*, *AGL*, *GBE1*, *PYGL*, *PHKB*, *PGAM2*, and *PRKAG2* were detected to be responsible for the onset of the clinical symptoms.”

now reads:

“A total of the 14 pediatric patients were admitted to our hospital and referred for molecular genetic testing using TGS. Seven genes namely *SLC37A4*, *AGL*, *GBE1*, *PYGL*, *PHKB*, *PGAM2*, and *PRKAG2* were detected to be responsible for the onset of the clinical symptoms.”

The original Article has been corrected.

