# Supplementary material for: Clinical and genetic spectrum of glycogen storage disease in Iranian population using targeted gene sequencing
Source: Sci Rep. 2021 Mar 29;11:7040. doi: 10.1038/s41598-021-86338-4 (PMC8007705; doi:10.1038/s41598-021-86338-4)
Supplement: Supplementary file 1 — Supplementary Information. [file 41598_2021_86338_MOESM1_ESM.pdf]

Clinical and Genetic Spectrum of Glycogen Storage Disease in Iranian Population using Targeted Gene Sequencing

Zahra Beyzaei PhD [1], Fatih Ezgu MD [2], Bita Geramizadeh MD[1,3*], Mohammad Hadi Imanieh MD[4], Mahmoud Haghighat MD[4], Seyed Mohsen Dehghani MD[4], Naser Honar MD[4], Mojgan Zahmatkeshan MD[4,5], Amirreza Jassbi PhD [6] , Marjan Mahboubifar PhD[6], Alireza Alborzi MPH[7]

[1]Shiraz Transplant Research Center (STRC), Shiraz University of Medical Sciences, Shiraz, Iran.

[2]Department of Pediatric Metabolic Disorders and Pediatric Genetics, Gazi University Faculty of Medicine, Ankara, Turkey.

[3]Department of Pathology, Shiraz University of Medical Sciences, Shiraz, Iran.

[4]Gastroenterology and Hepatology Research Center, Shiraz University of Medical Sciences, Shiraz, Iran.

[5]Department of Pediatrics, Shiraz University of Medical Sciences, Shiraz, Iran.

[6]Medicinal and Natural Products Chemistry Research Center, Shiraz University of Medical Sciences, Shiraz, Iran.

[7]Student Research Committee, Shiraz University of Medical Sciences, Shiraz, Iran.

*Corresponding Author: Bita Geramizadeh, Professor of Pathology, Department of Pathology, Shiraz University of Medical Sciences, Shiraz, Iran. Postal Address: Khalili St., Research Tower, Seventh Floor, Shiraz Transplant Research Center (STRC), Shiraz, Iran. Call number: 98-71-36472915, E-mail address: geramib@gmail.com

Table S1. Confirming Variants in Next-Generation Sequencing Panel Testing by Sanger Sequencing

GSD Types	Genes	Transcript	GRCh37 location	Exon/Intron	cDNA location	Strand	NGS result	Sanger result
GSD Ib	*SLC37A4*	NM_001164279.2	chr11:118900056	Exon8	c.24	-	T>G	T>G
GSD Ib	*SLC37A4*	NM_001164279.2	chr11: 118898407	Exon6	c.337	-	C>T	C>T
GSD III	*AGL*	NM_000642.3	chr1:100336041	Exon6	c.753_756	+	delCAGA	delCAGA
GSD III	*AGL*	NM_000642.3	chr1:100336041	Exon6	c.753_756	+	delCAGA	delCAGA
GSD III	*AGL*	NM_000642.3	chr1:100342081	Exon11	c.1351_1355	+	delAAAGC	delAAAGC
GSD III	*AGL*	NM_000642.3	chr1:100379113	Exon30	c.3980	+	G>A	G>A
GSD IV	*GBE1*	NM_000158.4	chr3:81643169	Exon8	c.998	-	A>T	A>T
GSD IV	*GBE1*	NM_000158.4	chr3:81754616	Exon2	c.292	-	G>C	G>C
GSD VI	*PYGL*	NM_002863.5	chr14:51378453	Exon16	c.1964	-	A>G	A>G
GSD VI	*PYGL*	NM_002863.5	chr14:51410891	Exon1	c.229_231	-	delGAC	delGAC
GSD IXc	*PHKG2*	NM_000294.2	chr16:30762461	Exon3	c.130	+	C>T	C>T
GSD IXb	*PHKB*	NM_000293.3	chr16:47531367	Exon2	c.134	+	T>A	T>A
GSD IXb	*PHKB*	NM_000293.3	chr16:47628046	Exon11	c.1127-2	+	A>G	A>G
GSD IXb	*PHKB*	NM_000293.3	chr16:47727384	Exon28	c.2840	+	A>G	A>G
GSD X	*PGAM2*	NM_000290.4	chr7:44105115	Exon1	c.14	-	G>A	G>A
GSD heart, lethal congenital	*PRKAG2*	NM_001040633.1	chr7:151329185	Exon5	c.592	-	A>T	A>T
